# Acceleration of a trailing positron bunch in a plasma wakefield accelerator

**DOI:** 10.1038/s41598-017-14524-4

**Published:** 2017-10-27

**Authors:** A. Doche, C. Beekman, S. Corde, J. M. Allen, C. I. Clarke, J. Frederico, S. J. Gessner, S. Z. Green, M. J. Hogan, B. O’Shea, V. Yakimenko, W. An, C. E. Clayton, C. Joshi, K. A. Marsh, W. B. Mori, N. Vafaei-Najafabadi, M. D. Litos, E. Adli, C. A. Lindstrøm, W. Lu

**Affiliations:** 1 LOA, ENSTA ParisTech, CNRS, Ecole Polytechnique, Universite Paris-Saclay, 91762 Palaiseau, France; 2 SLAC National Accelerator Laboratory, Menlo Park, CA 94025 USA; 3 University of California Los Angeles, Los Angeles, CA 90095 USA; 4 University of Colorado Boulder, Boulder, CO 80309 USA; 5 Department of Physics, University of Oslo, 0316 Oslo, Norway; 6 Department of Engineering Physics, Tsinghua University, Beijing, 10084 China

## Abstract

High gradients of energy gain and high energy efficiency are necessary parameters for compact, cost-efficient and high-energy particle colliders. Plasma Wakefield Accelerators (PWFA) offer both, making them attractive candidates for next-generation colliders. In these devices, a charge-density plasma wave is excited by an ultra-relativistic bunch of charged particles (the drive bunch). The energy in the wave can be extracted by a second bunch (the trailing bunch), as this bunch propagates in the wake of the drive bunch. While a trailing electron bunch was accelerated in a plasma with more than a gigaelectronvolt of energy gain, accelerating a trailing positron bunch in a plasma is much more challenging as the plasma response can be asymmetric for positrons and electrons. We report the demonstration of the energy gain by a distinct trailing positron bunch in a plasma wakefield accelerator, spanning nonlinear to quasi-linear regimes, and unveil the beam loading process underlying the accelerator energy efficiency. A positron bunch is used to drive the plasma wake in the experiment, though the quasi-linear wake structure could as easily be formed by an electron bunch or a laser driver. The results thus mark the first acceleration of a distinct positron bunch in plasma-based particle accelerators.

## Introduction

Particle acceleration based on radio-frequency cavities is the technology used in today’s particle collider facilities. Because of the limited electric field that can be sustained in radio-frequency cavities, below 100 MV m^−1^, the length and the cost of such machines become prohibitively large for particles colliding at energies in the teraelectronvolt (TeV) range. For example, the proposed International Linear Collider (an electron-positron collider) has a 31-km length for a centre-of-mass energy of 500 gigaelectronvolt (GeV)^1^. Plasma-based particle accelerators^2^ provide large acceleration gradients, which can exceed 100 GeV m^−1^ ^3^, and high energy efficiency^4,5^, and therefore open the prospect of reaching much higher particle energies while keeping a similar accelerator footprint. Although plasma acceleration of individual electrons^3,6–9^ and positrons^10,11^ with broad energy spectra has been demonstrated, the efficient acceleration of a distinct trailing bunch of electrons with a narrow energy spread was only recently achieved^4^. However the acceleration of positrons, the antimatter counterpart of electrons, is imperative for applying the plasma acceleration technique to electron-positron colliders^12^. A multi-GeV acceleration of positrons was accomplished^13^ by using a highly nonlinear regime of PWFA where a single bunch of positrons interacts with the plasma, with particles in the front transferring their energy to the particles in the rear of the bunch. While this strategy of energy transfer within a single bunch is promising for the realization of a plasma-based single-stage energy booster to an existing electron-positron collider^14^, the staging of PWFA accelerator modules requires the ability to accelerate a distinct trailing bunch of positrons in a plasma. Although multiple technological limitations still need to be overcome, the work presented here accomplishes one important step towards realizing a plasma-based collider.

## Results

### Experimental set-up and parameters

**Figure 1:**
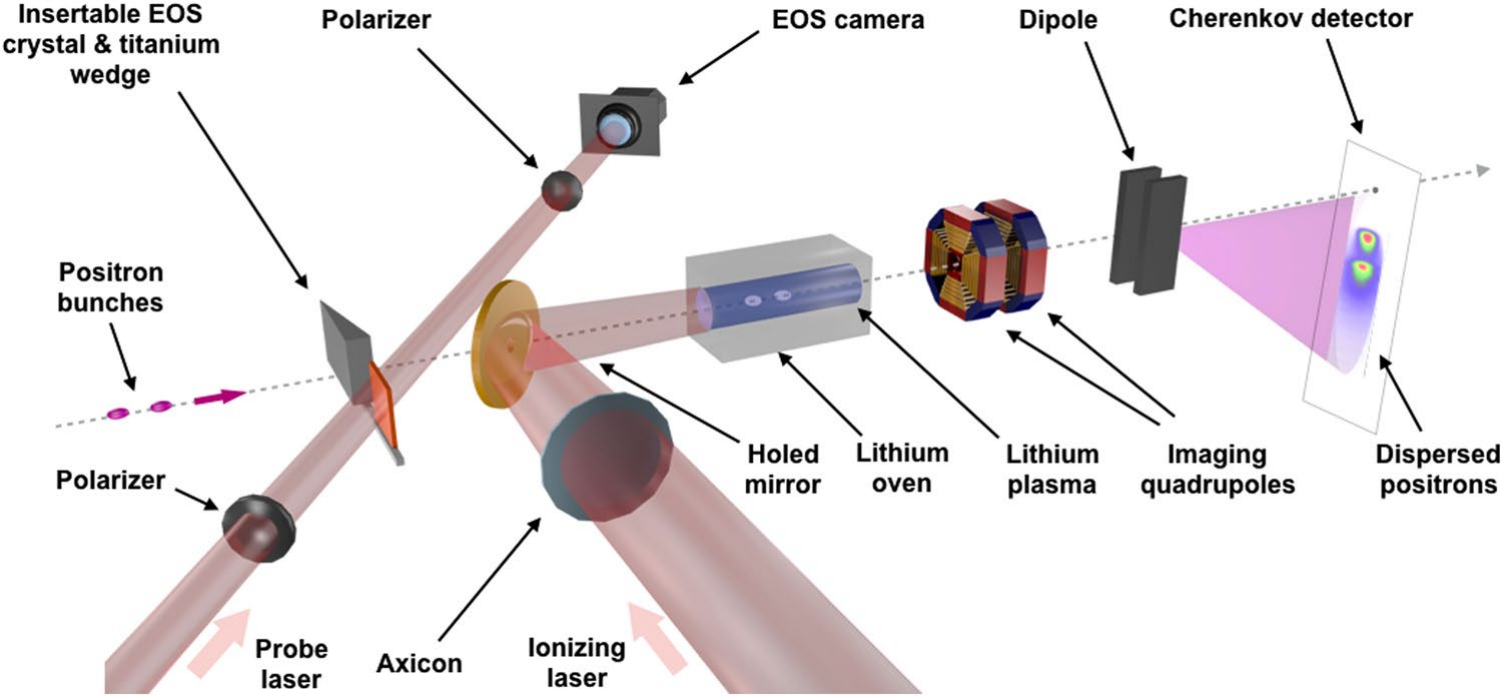
Experimental set-up. An intense laser is focused by an axicon in a lithium oven, and creates a uniform plasma by ionization of the first electron of lithium. Two positron bunches, a drive bunch at a central energy of 20.55 GeV, and a trailing bunch at a central energy of 20.05 GeV about 100 *μ*m behind, are sent into the plasma. An electro-optic sampler (EOS) and a titanium wedge can be inserted in the beamline. A Cherenkov imaging spectrometer is set up to measure the energy spectrum of the positrons after the interaction with the plasma.

The acceleration of a trailing positron bunch reported here was demonstrated in an experiment conducted at the Facility for Advanced Accelerator Experimental Tests (FACET)^15^ at the SLAC National Accelerator Laboratory. This facility provides a 20.35 GeV positron beam to the experimental area, where the beam is reshaped (see Methods) into a two-bunch longitudinal profile. Bunches have central energies of 20.55 GeV for the drive bunch and 20.05 GeV for the trailing bunch, and are longitudinally separated by about 100 *μ*m, the trailing bunch propagating behind the drive. They are focused to *x* and *y* root-mean-square (r.m.s.) beam sizes of 35 *μ*m × 25 *μ*m by the final focusing system. The drive contains 480 pC of charge and has a r.m.s. bunch length of *σ*_z_ = 30 *μ*m, and the trailing bunch contains 260 pC of charge and has a r.m.s. bunch length of *σ*_z_ = 30 *μ*m. The plasma source consists of a 1.3-m-long column of lithium vapour^16^ (see Methods), pre-ionized by a femtosecond laser focused on a line by an axicon optic as depicted in Fig. 1 ^17^. The plasma density was set to 1×10^16^ cm^−3^, corresponding to a plasma wavelength of 330 *μ*m. A diagnostic based on an electro-optic sampling technique (EOS)^18^ was used to provide a quantitative description of the beam longitudinal profile (see Methods). After interacting with the plasma, the exiting positron bunches were characterized by an energy spectrometer which consists of a quadrupole doublet for imaging, a dispersive dipole and a Cherenkov detector^19^ (see Methods).

### Experimental evidence

**Figure 2:**
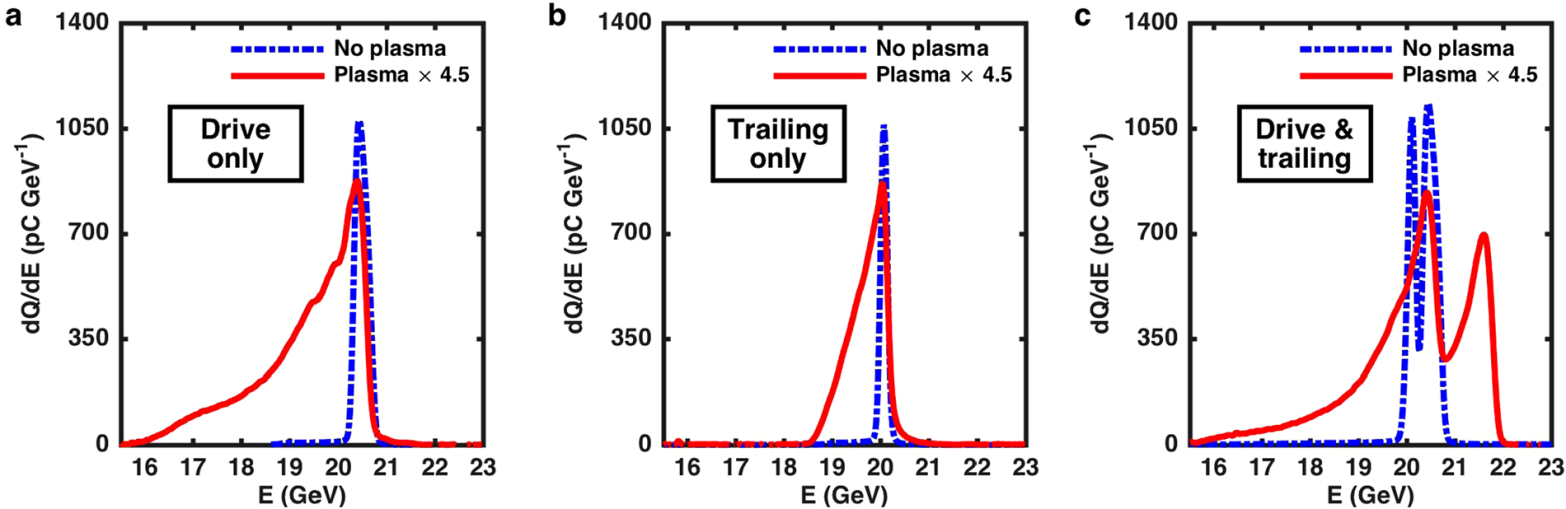
Evidence of acceleration of a positron bunch. Positron energy spectra measured by the Cherenkov energy spectrometer with (red solid line) and without plasma (ionizing laser off, blue dash-dotted line). In (**a)** only the drive bunch is sent, in (**b)** only the trailing bunch is sent, and in (**c**) both the drive and the trailing bunches are sent through the lithium oven, in which case the trailing bunch is accelerated from 20.05 GeV to above 21 GeV by the plasma wake. The plasma data is multiplied by 4.5 in (**a**–**c)**. The spectrum **c** with plasma is one of the 160 spectra from the dataset used in Fig. 3.

The demonstration of the acceleration of the distinct trailing positron bunch is depicted in Fig. 2. In the drive-trailing bunch configuration, it was possible to send only the drive, or only the trailing bunch, or both bunches, either through the laser-ionized plasma or the unionized lithium vapour. When only the drive bunch is sent into the plasma, as shown in Fig. 2a, positrons lose up to a few GeV of energy in the plasma by exciting a plasma wave in their wake. The same phenomena is observed when only the trailing bunch is sent, as shown in Fig. 2b, but with particles losing less energy because of the smaller charge of this bunch. In both cases, the bunch of particles sent into the plasma interacts with the electrons in the medium and transfers energy to a plasma wake, resulting in a decrease of the energy of the particles of the bunch. When both bunches are sent into the plasma, a peaked accelerated spectrum is observed (see Fig. 2c). At ultra-relativistic speeds ($v\simeq c$) and over the meter scale of the plasma, there is no relative motion between the two bunches. Causality implies that the trailing bunch cannot influence the drive bunch, as the drive is located ahead of the trailing bunch. Only the drive bunch can influence the trailing bunch through wakefield excitation. As can be seen in Fig. 2a, no drive particles are observed above 21 GeV. Therefore particles above 21 GeV in Fig. 2c originate from the trailing positron bunch, and their energy gain results from the presence of the drive bunch ahead of them. That is, the trailing positrons are accelerated by the plasma wave excited by the drive bunch. To determine the charge, the peak energy and the energy spread of the accelerated particles, an asymmetric Gaussian function is fitted to the measured spectrum shown in Fig. 2c above 20.8 GeV (upper limit of the drive bunch). The r.m.s. energy spread associated with the fit of the peak is 1.0%, while the charge is 85 pC and the peak energy is 21.5 GeV. This accelerated charge compares favorably to the state-of-the-art in PWFA^4^. The initial trailing bunch energy being at 20.05 GeV, this corresponds to an energy gain of 1.45 GeV and, for a plasma length of 1.3 m, to an accelerating energy gradient of 1.12 GeV m^−1^. The wake-to-bunch energy extraction efficiency, defined as the total amount of energy gained by all the particles in the trailing bunch with final energy above 20.8 GeV relative to the total amount of energy lost by all the particles in the drive bunch with final energy below 19.9 GeV, is estimated to be 40%, similar to previous experimental results^4,13^. This parameter describes the fraction of the energy transferred to the plasma wake that is extracted by the trailing bunch^4,13^.

### Beam loading phenomenon

In the scenario described above, a substantial number of trailing positrons are extracting energy from the plasma wave as they are accelerated. This energy extraction implies a decrease of the longitudinal electric field amplitude of the wake, which may be flattened under optimal conditions. This so-called beam-loading phenomenon has been extensively explored for the case of electrons, in both linear^20^ and nonlinear regimes^21–23^. In the experiment we report here, the wake is set up by a first bunch of particles, the drive bunch. The plasma electrons are set into motion radially by the attractive force of this drive bunch, made for technical reasons of positrons. Compared to the single-bunch conditions used in ref.^13^, the number of particles in the drive bunch is as low as 3 × 10^9^ particles (a much lower driving force) and the plasma density is set to 10^16^ cm^−3^ (a much lower restoring force). As a consequence, the inward flowing electrons cross the propagation axis behind the drive bunch and the driver wake is sampled by a distinct trailing bunch of positrons placed at the appropriate distance behind the drive bunch. In the present case, the trailing bunch is located 100 *μ*m behind the drive bunch. In principle, thanks to the mechanism described above, the driver could also be an electron bunch or a laser pulse. Recent theoretical results have suggested the use of ring-shaped electron drivers^24^ or Laguerre-Gaussian laser pulses^25^ to facilitate positron wakefield acceleration in the nonlinear regime.

**Figure 3:**
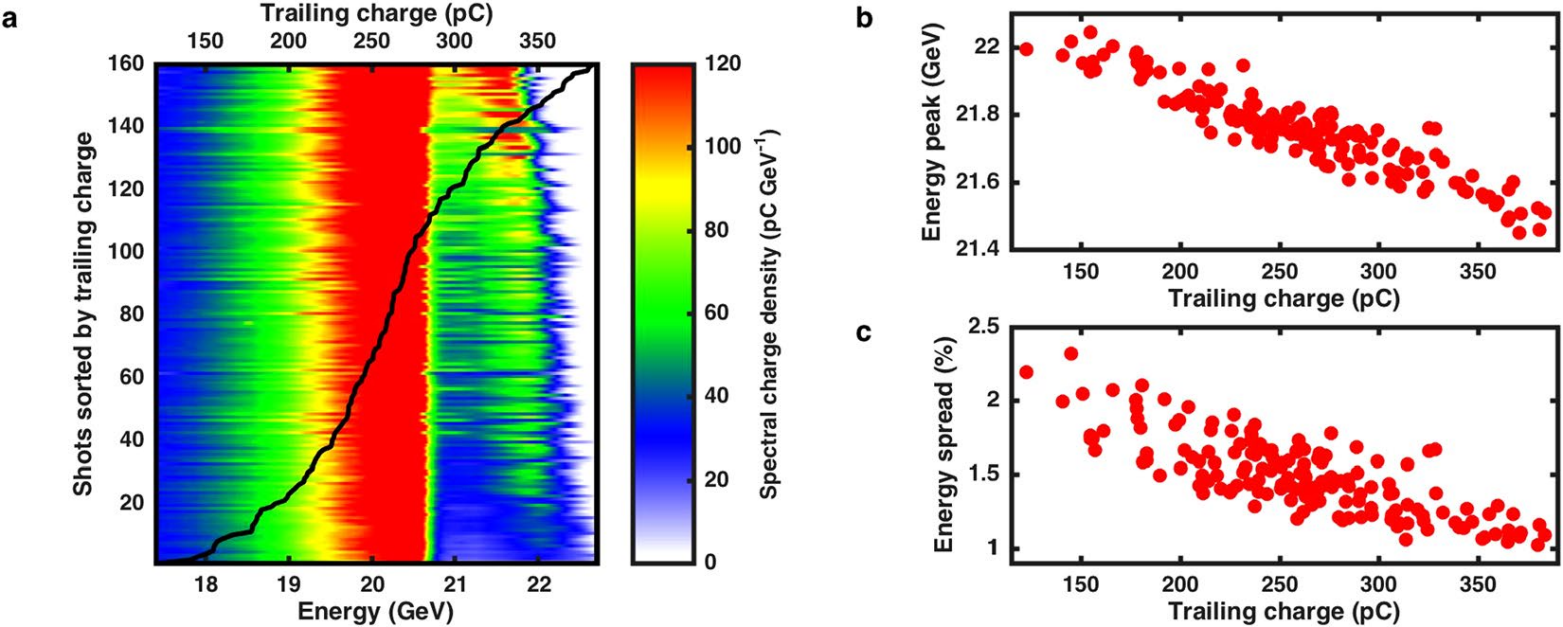
Energy spectra and evidence for the beam-loading phenomenon. (**a)** A stack of 160 energy spectra of the positron bunches measured after interaction with the plasma. Each horizontal line represents a single spectrum (see the comments on Fig. 2c). The shots are sorted by increasing trailing bunch charge (black line). The color axis represents the spectral charge density in pC GeV^−1^. (**b**) Energy of the accelerated peak as a function of the trailing bunch charge. (**c)** The r.m.s. energy spread of the accelerated peak (obtained from the asymmetric Gaussian fit) as a function of the trailing bunch charge.

The beam-loading phenomenon was evidenced in the experiment by correlating the energy gain and energy spread of the trailing bunch with the charge of this bunch before it enters the plasma: the incoming trailing charge. The natural shot-to-shot variation of the trailing bunch charge was used for the study of beam loading and the results are shown in Fig. 3. The full data set consists of 160 beam shots. An average distance between the drive and trailing bunches of 100 *μ*m was measured with the EOS diagnostic and the trailing charge ranged from 100 to 400 pC with a mean value of 260 pC and a standard deviation of 55 pC. The average energy of the accelerated peak is 21.75 GeV, corresponding to a 1.70 GeV energy gain, and the average r.m.s. energy spread is 1.5% (obtained from the asymmetric Gaussian fit). On the waterfall plot depicted in Fig. 3a, energy spectra are sorted by increasing trailing bunch charge. Accelerated particles appear on the right, above 20.8 GeV (upper limit of the drive bunch). Two correlations are observed: the peak energy and the energy spread of the accelerated particles decrease when trailing bunch charge increases. The quantitative plots of the correlations with the trailing bunch charge are represented in Fig. 3b (for the energy of the accelerated peak) and Fig. 3c (for the r.m.s. energy spread obtained from the fit). As the trailing bunch charge is increased to its maximum value in the experiment, the energy spread decreases from about 2% down to 1% while the energy gain decreases from about 1.95 GeV to 1.45 GeV. These correlations imply that the maximum longitudinal electric field is reduced as the charge of the trailing bunch is increased, which would result in a flattening of the electric field, consistent with the observed decrease of the energy spread.

### Effect of beam emittance on plasma wakefields

The propagation and evolution of a positron bunch in a plasma strongly depends on its normalized *x* and *y* emittances, which quantify the area the bunch occupies in the transverse phase spaces (*x*,*p*_*x*_) and (*y*,*p*_*y*_) respectively. Due to the transverse force from the excited plasma wakefield, small emittance bunches will evolve and reach a small transverse size in a plasma, and possibly become much denser than the plasma $({n}_{{\rm{beam}}}\gg {n}_{{\rm{plasma}}})$, thereby entering the nonlinear regime of PWFA. In contrast, for large emittances, the focusing force can be balanced by the emittance term in the beam envelope equation^26^, and the transverse size reached after evolution in the plasma is larger, leading to a more linear wake. By varying the bunch emittance, the regime of wakefield excitation can therefore be controlled. In the experiment, the beam emittance was increased gradually using an insertable titanium wedge located 1.65 m upstream of the plasma entrance, as depicted in Fig. 1. The thickness of the titanium wedge in the beam path can vary from 20 to 400 *μ*m. By doing so, the emittance is increased due to multiple small-angle scattering events of the positrons in the titanium. Given the initial *x* and *y* emittances of 100 *μ*m × 10 *μ*m, the titanium wedge can increase the emittance by up to three times in *x* and seven times in *y*. Because of the geometrical constraints of the experiment, the titanium wedge cannot be located closer to the plasma entrance, and as a result the emittance increase comes with an increase of the beam sizes at the plasma entrance. The initial *x* and *y* r.m.s. beam sizes are 35 *μ*m × 25 *μ*m without titanium and increase up to 101 *μ*m × 98 *μ*m for the highest titanium thickness, which can also contribute to the transition towards a more linear regime as the titanium thickness is increased. Note that the smaller *y* emittance (compared to the *x* emittance) can lead to a smaller transverse extent for the plasma wake in that direction and to a slight difference in the shape of the plasma wakefields.

**Figure 4:**
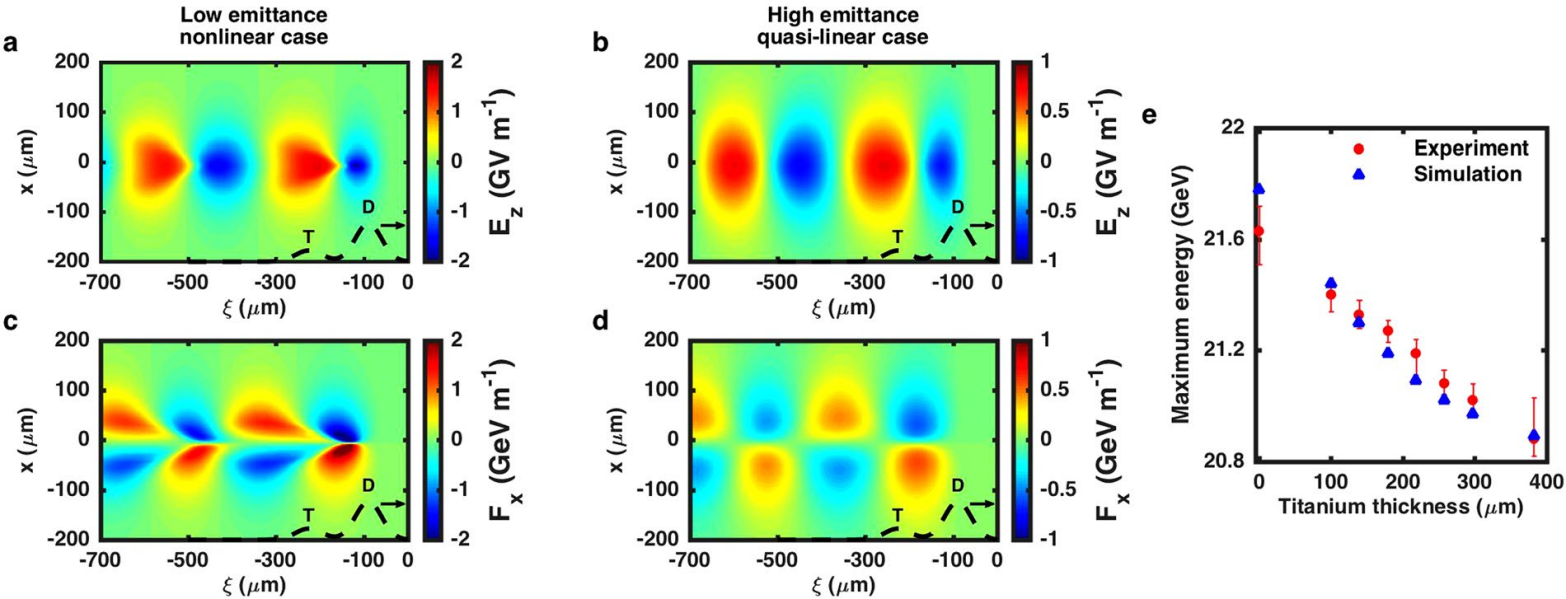
Simulated nonlinear and quasi-linear wakefields, and maximum energy. (**a**,**b**) Longitudinal electric field E_z_ in the plasma wake for the low emittance case (no titanium) with *x* and *y* emittances of 100 *μ*m × 10 *μ*m (**a**) and for the high emittance case (297 *μ*m of titanium) with *x* and *y* emittances of 270 *μ*m × 60 *μ*m (**b**). (**c**,**d**) Transverse force F_x_ $\simeq$ e(E_x_ − cB_y_) experienced by the positrons in the plasma wake for the low emittance case (**c**) and for the high emittance case (**d**). **e**, Maximum energy (defined as the energy where the accelerated spectrum crosses the 10 pC GeV^−1^ threshold) as a function of the titanium thickness, in the experiment and in simulations. As the most important error on the maximum energy arises from the sensitivity to the threshold, the error bars depict the effect of multiplying and dividing the threshold by 1.5. In (**a**–**d)** *x* is the transverse coordinate, ξ = z − ct is the dimension parallel to motion, and the bunches are propagating to the right (direction indicated by the black arrows). Black dashed lines represent the current profile and depict the positions of the drive (D) and trailing (T) bunches in the wakefield.

The plasma wakefields and their evolution are computed using the particle-in-cell code QuickPIC^27,28^ with beam and plasma parameters similar to those of the experiment (see Methods). They evolve in the density upramp and the first ten centimeters of the density plateau and then reach a quasi-steady state with negligible evolution. Figure 4a–d display the shape of the plasma wakefields in the middle of the plasma (at *z* = 72.5 cm, *i.e*. after the quasi-steady state is reached), for emittances of 100 *μ*m × 10 *μ*m (no titanium) and for emittances of 270 *μ*m × 60 *μ*m (297 *μ*m of titanium). In the low emittance case, the wake has a strong nonlinear structure and *n*_beam_/*n*_plasma_ = 14.2 for the drive bunch in the plasma. The longitudinal fields show very steep and asymmetric gradients (Fig. 4a) and the shape of the transverse force (Fig. 4c) strongly depends on the longitudinal coordinate *ξ* = *z*−*ct*, that is the transverse wakefield is non-separable (it cannot be written as product of a function of *x* and a function of $\xi$). The plasma wakefields in the $\xi y$ plane also shows a nonlinear structure. In this regime, the accelerated bunch takes a specific arrowhead shape^13^ due to the shape of the pseudo-potential $\psi$ defined implicitly by ${{\bf{F}}}_{\perp }=-{\nabla }_{\perp }e\psi$. The pseudo-potential well confines the trailing bunch transversely and allows for its acceleration over the full plasma length. The two slightly off-axis minimums of the pseudo-potential well lead to a high trailing bunch density around these two minimums and to the arrowhead shape. In the high emittance case, Fig. 4b and d show a quasi-linear wakefield structure^29^ and ${n}_{{\rm{beam}}}/{n}_{{\rm{plasma}}}=1.2$ for the drive bunch in the plasma. Although the maximum longitudinal electric field *E*_*z*_ is reduced from about 1.6 GeV m^−1^ to 0.8 GeV m^−1^, the wakefield is more regular and the transverse force takes a separable and sinusoidal form, characteristic of the linear regime of PWFA. This quasi-linear regime is interesting due to its more symmetrical properties for electrons and positrons^29^, and its wakefield regularity may be an advantage for preserving positron beam quality during acceleration to high energies.

In the experiment, acceleration of positrons from the trailing bunch was observed on the spectrometer over the whole range of the achievable titanium thicknesses. When the emittance was progressively increased, the interaction of the beam with the plasma became accordingly weaker, and the energy of the accelerated bunch became close to the initial drive bunch energy, leading to a much less pronounced spectral peak on the spectrometer. Figure 4e shows the maximum energy of the trailing bunch, defined as the energy at which the accelerated spectrum crosses the 10 pC GeV^−1^ threshold, as a function of the titanium thickness. It shows that, in the experiment, the maximum trailing bunch energy decreases when the titanium thickness is increased, as expected from a transition from a nonlinear to a quasi-linear regime. These experimental results show similar trends with particle-in-cell simulations (also shown in Fig. 4e), and thus demonstrate the acceleration of a trailing positron bunch spanning nonlinear to quasi-linear regimes^29^ of plasma wakefield acceleration.

## Discussion

In conclusion, a distinct trailing positron bunch was successfully accelerated in a plasma wakefield accelerator with an energy gradient exceeding a gigaelectronvolt-per-meter. This result represents a significant step forward for the long-term prospect of the realization of a high-energy electron-positron collider based on plasma acceleration technology, and opens promising paths for positron acceleration in nonlinear and quasi-linear regimes of plasma wakefield acceleration.

## Methods

### Positron beam and plasma parameters

The FACET facility at SLAC National Accelerator Laboratory delivers to the experimental area a 20.35 ± 0.05 GeV positron bunch. The positron bunch originates from an electron beam produced by the linear accelerator (linac) that hits a tungsten alloy target, which results in electromagnetic cascades inside it and leads to electron-positron pair production. Positrons exiting the target are captured and transported back to the start of the linac to be accelerated to a final energy of 20.35 ± 0.05 GeV. The final focusing system, consisting of five quadrupole magnets, focuses the beam down to r.m.s. spot sizes of 35 ± 10  *μ*m in *x* and 25 ± 10 *μ*m in *y*. The normalized emittances of the beam in transverse directions were 100 ± 30 *μ*m in *x* and 100 ± 30 *μ*m in *y*. The plasma source used in this experiment was a column of lithium vapour^16^, pre-ionized by a femtosecond laser^17^ and confined by a helium buffer on both ends. The plasma was 1.30 m long (FWHM), composed of a density plateau of length 1.15 m with 15-cm-long linear density ramps on both ends. The lithium atomic density in the plateau region was set to 10^16^ cm^−3^. The laser was set up to ionize the lithium vapour around 100 ps before the positron beam passes through it, with a mean energy of 120 mJ. The laser duration was 200 fs (FWHM), and it was focused on a line through the entire length of lithium vapour using a 0.6 degree axicon.

### Generation of two-bunch beam structure

The longitudinal shaping of the beam requires further manipulation to reach a two-bunch structure. Shaping takes place in a “W-shaped” chicane. At the chicane entrance, the longitudinal positions of positrons along the bunch are related to their energy, with highest energies at the front and lowest at the rear of the bunch. The first dipole of the chicane adds a correlation between energy *E* and transverse position *x* to the bunch. At this point a tantalum bar called “œnotch collimator system” is placed in the path to remove central energy particles from the bunch, a process that results also in the loss of a substantive part of the total charge. This cut of the central energy particles leads to the formation of a two-bunch structure, with the high energy bunch centered at 20.05 ± 0.05 GeV, and the low energy bunch centered at 20.05 ± 0.05 GeV. At the end of the chicane, the correlation (*E*, *x*) is removed. The chicane does not fully compress, so as to retain a correlation between energy and longitudinal position, with higher energy positrons still being at the front and lower energy positrons at the rear (under-compression). After the whole process, the two-bunch structure has a high energy drive bunch with *σ*_*z*_ = 30 ± 10 *μ*m and a low energy trailing bunch with *σ*_*z*_ = 40 ± 10 *μ*m, separated by 100 ± 10 *μ*m for the data of Figs 2 and 3 and by 165 ±10 *μ*m for the data of Fig. 4. The average charge of the drive bunch is 480 ± 10 pC, and the average trailing bunch charge is 260 ± 10 pC for the data of Figs 2 and 3 and 140 ± 10 pC for the data of Fig. 4.

The longitudinal current-profile diagnostic relies on an electro-optic sampling system on the scheme described by Litos *et al*.^30^. Figure 1 pictures its inclusion into the plasma experiment. The setup consists of a 50 *μ*m-thick GaP crystal standing a millimeter below the beam path and of a probe laser beam passing through the crystal at an angle, with an adjustable delay relative to the positron beam passage. The GaP electro-optic crystal birefringence is modified by the transient, sliced shaped electric field of the positron bunches^18^. The femtosecond probe laser pulse passes through the crystal to sample its instantaneous local properties. Birefringence induced by the positron beam in the crystal result in a relative phase delay between the two orthogonal components of the laser. Two polarizers are used, a first one before the crystal, with a main direction at a 45 degree angle relative to the crystal axis and a second one, located before the detector and aligned for laser extinction in the absence of a positron beam. A higher laser signal on the detector is observed in the areas in which birefringence was modified by the positron beam. Those areas correspond to a perfect synchronicity between beam field and probe pulse. Because the intersection time varies along the transverse position of the crystal and the probe laser, the temporal profile of the positron beam can be retrieved with a single measurement, with a temporal resolution of about 150 fs. The longitudinal separation between the drive and trailing bunches can be modified experimentally by varying the arrival time of the positron beam in the linac and the EOS diagnostic monitors the resultant change.

### Cherenkov energy spectrometer

The beam energy spectrum after interaction in the plasma is measured as depicted in Fig. 1. Two quadrupole magnets image–for a given positron energy *E*_image_–the exit of the plasma onto the air-gap Cherenkov detector^19^. For the data of Figs 2 and 3, *E*_image_ = 21.35 GeV, and for the data of Fig. 4, *E*_image_ = 20.85 GeV. A dipole set up on the beam path, after the quadrupoles, deflects the particles vertically onto the detector and provides a correlation between the particle vertical position and its energy. The energy resolution depends on the finite vertical beam size at the detector and on the detector resolution itself, and is of the order of 50 MeV for energies near *E*_image_. A camera records the Cherenkov light emitted by the particles in the 5-cm-long air gap.

### Particle-in-cell simulations

Simulation results shown in Fig. 4 were performed with the particle-in-cell QuickPIC code^27,28^. QuickPIC is a kinetic simulation code working in the quasi-static approximation, which assumes that the particle beam is evolving slowly compared to the time scale of the plasma response. The simulation box moves at the speed of light in the beam propagation direction and its coordinate system is *x*, *y* (transverse coordinates) and *ξ* = *z* − *ct* (longitudinal coordinate). The box has a size of 400 × 400 × 700 *μ*m^3^ (in *x*, *y* and *ξ*) and consists of 512 × 512 × 1024 cells. In the simulations, the drive and trailing bunches are initialized with Gaussian profiles in all dimensions and with r.m.s. spot sizes of *σ*_*x*_ = 35 *μ*m and *σ*_*y*_ = 25 *μ*m, and r.m.s. bunch lengths of *σ*_*z*_ = 30 *μ*m (for the drive bunch) and *σ*_*z*_ = 40 *μ*m (for the trailing bunch). The longitudinal separation between the drive and trailing bunches is 135 *μ*m. Both bunches in the simulation have zero initial energy spread and their central energy is 20.55 GeV (for the drive bunch) and 20.05 GeV (for the trailing bunch). The charge of the bunches is chosen to be 50% of the charge measured experimentally, as this yields better agreement with the experimental data. As previously described in ref^4^. this adjustment is justified by the presence of transverse tails in the positron charge distribution that do not take part in the interaction. Finally, the scattering angle induced by the titanium wedge system is calculated from the titanium thickness, and the new Twiss parameters and emittances of the beam at the entrance of the plasma are computed and used for the initialization of the beam in the QuickPIC simulation.

### Data availability

The data that support the findings of this study are available from the corresponding author upon request.
